# A novel *de novo* missense variant in *ASH1L* associated with mild autism spectrum disorder and an uneven cognitive profile: a case report

**DOI:** 10.1186/s13256-025-05675-4

**Published:** 2025-11-25

**Authors:** Otabek Pulatov, William Nguyen, Diego Alvarez Vega, Romina Barros

**Affiliations:** 1 New York University Grossman Long Island School of Medicine, Mineola, NY USA; 2 Department of Developmental Pediatrics, NYU Langone Health, Long Island, Mineola, NY USA

**Keywords:** *ASH1L*, Autism spectrum disorder, Intellectual disability, Whole genome sequencing, Neurodevelopmental disorders

## Abstract

**Background:**

*ASH1L*-related intellectual developmental disorder represents an emerging neurodevelopmental syndrome with significant phenotypic heterogeneity (Cordova *et al*. in *Genes* (Basel). 15(4):423, 2024). Comprehensive genomic analysis demonstrates superior diagnostic yield compared with targeted approaches in complex neurodevelopmental presentations (Srivastava *et al*. in *Genet Med*. 21(11):2413–2421, 2019).

**Case presentation:**

This report describes a 6-year-old Central Asian (Uzbek) male patient with a history of global developmental delay who was diagnosed with mild autism spectrum disorder, attention-deficit/hyperactivity disorder, and a developmental expressive language disorder. Neuropsychological assessment revealed an uneven cognitive profile with average verbal abilities but below-average nonverbal reasoning. After uninformative targeted genetic panels, trio whole-genome sequencing identified a novel *de novo* heterozygous missense variant in *ASH1L* c.4043A > G (*p*.Lys1348Arg). This variant, absent in population databases, was classified as a variant of uncertain significance. However, *in silico* analysis predicted this variant to be probably damaging, and therefore, it emerged as the strongest candidate to explain the patient’s phenotype.

**Conclusion:**

This case expands the known phenotypic spectrum of *ASH1L*-related disorders, demonstrating that a *de novo* missense variant can be associated with a milder neurodevelopmental phenotype, including borderline-to-average intellectual ability. These findings challenge suggestions that missense variants uniformly lead to more severe outcomes and underscores the importance of comprehensive genomic and deep clinical characterization to refine our understanding of gene–disease relationships.

## Introduction

ASH1L (ASH1-like histone lysine methyltransferase) functions as a critical chromatin-modifying enzyme essential for neurodevelopmental processes [1]. Located on chromosome 1q22, *ASH1L* gene encodes a 2969-amino acid protein containing multiple functional domains including SET methyltransferase, AWS, and PHD finger domains [2]. The protein catalyzes histone H3 lysine 36 trimethylation, regulating transcriptional activation during cortical development and synaptic plasticity [3].

Heterozygous pathogenic variants in ASH1L cause intellectual developmental disorder, autosomal dominant 52 (IDD52; OMIM #617,796), a clinically heterogeneous neurodevelopmental disorder. Core features include developmental delay, intellectual disability (ID) ranging from mild to severe, and a high incidence of autism spectrum disorder (ASD) and attention-deficit/hyperactivity disorder (ADHD). Significant speech delay, hypotonia, and motor deficits are also common [4]. Most pathogenic *ASH1L* variants are loss-of-function (LoF), causing disease via haploinsufficiency [5]. Missense variants are less frequent, and an emerging hypothesis suggests they may cause more severe phenotypes, possibly through a dominant-negative effect, particularly when located in critical functional domains.

Whole genome sequencing increasingly demonstrates superior diagnostic yield compared with conventional genetic testing in neurodevelopmental disorders [6]. Meta-analyses indicate 41% diagnostic rates for genome sequencing versus 24% for targeted approaches, with particular advantages in identifying *de novo* variants in emerging disease genes [7]. The American College of Medical Genetics now recommends comprehensive genomic testing as first-tier evaluation for patients with complex neurodevelopmental presentations [8].

This report adheres to the case report (CARE) guidelines to ensure accurate and transparent reporting.

## Case presentation

### Patient history

The proband is a 6-year-old male of Central Asian descent, the first child of healthy, non-consanguineous parents, who was referred for a second opinion regarding a prior autism diagnosis. Family history was negative for neurodevelopmental disorders, intellectual disability, or known genetic conditions across three generations. His perinatal history was unremarkable except for mild hypotonia. Key developmental milestones were delayed: he sat independently at 8 months, walked at 20 months, and spoke his first words around 2 years of age.

Early developmental milestones demonstrated delays in social communication, with his first words being at 22 months and two-word phrases at 30 months. Autism spectrum disorder was diagnosed at 24 months using ADOS-2 assessment, meeting criteria in both social affect (calibrated severity score 8) and restricted repetitive behavior (calibrated severity score 7) domains with total score 18 in autism range [9]. ADHD was diagnosed by a pediatric neurologist at the age of 5.5 years.

### Physical, neurological, and neurodevelopmental examination

On examination, his growth parameters were within normal limits. Subtle dysmorphic features included an elongated face with protruding, mildly asymmetric ears and two café-au-lait macules. Neurological examination revealed mild developmental coordination disorder. Hyperactivity and impulsivity were noted, consistent with his established ADHD diagnosis.

A comprehensive neuropsychological evaluation revealed a pattern of general average-range abilities punctuated by specific weaknesses (Table 1). Cognitive testing (Kaufman Brief Intelligence Test, 2nd Ed. [KBIT-2R]) [10] showed a statistically significant discrepancy (*p* < 0.01) between his average verbal skills and below-average nonverbal reasoning. Academic skills (Wide Range Achievement Test, 5th Ed. [WRAT-5]) [11] were average to high-average. Language testing (Oral and Written Language Scales, 2nd Ed [OWLS-II]) [12] confirmed a developmental expressive language disorder, with age-appropriate receptive skills but below-average expressive abilities. Visual-motor integration (Beery–Buktenica Developmental Test of Visual-Motor Integration [Beery VMI]) [13] was low-average, attributed to fine-motor deficits rather than perceptual issues. Behavioral rating scales (Vanderbilt, Child Behavior Checklist [CBCL], and Teacher’s Report Form [TRF]) [14] confirmed ADHD symptoms and noted teacher concerns for anxiety and obsessive–compulsive problems. Despite a low score on the CARS-2 HF (Childhood Autism Rating Scale, 2nd Ed., High Functioning) [15], a final clinical diagnosis of mild ASD was made on the basis of the longitudinal history and qualitative observations of social communication deficits.

**Table 1:** Summary of neuropsychological and behavioral assessment results

Assessment tool	Subtest/scale	SS/T	Percentile	Age equivalent	Qualitative interpretation
KBIT-2R	Verbal	Average SS	—	—	Average
	Nonverbal	Below-average SS	—	—	Below average
	*Verbal versus nonverbal discrepancy*	—	—	—	*Statistically significant (p* < *0.01)*
WRAT-5	Math computation	High-average SS	—	—	High average
	Word reading, spelling	Average SS	—	—	Average
OWLS-II	Listening comprehension	SS 99	47th	6 years 6 months	Average
	Oral expression	SS 82	12th	5 years 7 months	Below average
Beery VMI	Visual–motor integration	SS 88	21st	5 years 6 months	Low average
	Visual perception	SS 105	63rd	7 years 4 months	Average
CARS-2 HF	Total score	T 41	19th	—	Minimal to no symptoms of ASD
SRS-2	Parent form	T 62	—	—	Mild range
	Teacher form	T 57	—	—	Within normal limits
Vanderbilt	Parent form (inattention)	7/9 endorsed	—	—	Meets criteria for inattentive type
	Teacher form (hyperactivity)	8/9 endorsed	—	—	Meets criteria for combined type
CBCL (parent)	Attention problems	T > 65	93rd–97th	—	Borderline clinical
TRF (teacher)	Anxious/depressed	T > 70	> 97th	—	Clinical
	Thought problems	T > 70	> 97th	—	Clinical
	Obsessive–compulsive	T > 70	> 97th	—	Clinical

KBIT-2R = Kaufman Brief Intelligence Test, 2nd Ed.; WRAT-5 = Wide Range Achievement Test, 5th Ed.; OWLS-II = Oral and Written Language Scales, 2nd Ed.; Beery VMI = Beery-Buktenica Developmental Test of Visual-Motor Integration; CARS-2 HF = Childhood Autism Rating Scale, 2nd Ed., High Functioning; SRS-2 = Social Responsiveness Scale, 2nd Ed.; CBCL = Child Behavior Checklist; TRF = Teacher’s Report Form; SS, standard score; T, T-score

### Diagnostic genetic testing

Prior genetic testing, including a chromosomal microarray and a large 1038-gene panel at the age of 5 years, was uninformative; the panel did not include the *ASH1L* gene. Subsequent trio whole-genome sequencing (WGS) identified a heterozygous de novo* missense variant in ASH1L c.4043A* > *G (p.Lys1348Arg)* located in exon 20 (Table 2). The variant was absent from population databases including gnomAD v3.1 (allele frequency 0/251,406 alleles) and absent from ClinVar [16]. Sanger sequencing confirmed *de novo* occurrence with both parents testing negative. The variant affects a moderately conserved lysine residue located well outside the C-terminal SET methyltransferase domain, which is critical for the protein’s enzymatic function [3].

**Table 2:** Summary of key genetic findings

Gene	Variant (HGVS nomenclature)	Zygosity	Inheritance	Classification	Testing method	Relevance to phenotype
*ASH1L*	c.4043A > G (*p*.Lys1348Arg)	Heterozygous	De novo	VUS	WGS	Strongest candidate
*PDE4D*	c.2088_2096del (*p*.Ile697_Asp699del)	Heterozygous	De novo	VUS	WGS	Unlikely contributor (no phenotypic overlap)
*FMR1*	54 CGG repeats	Hemizygous	Maternal	Intermediate allele	Repeat expansion analysis	Incidental familial finding
*HIBCH*	c.469C > T (*p*.Arg157*)	Heterozygous	Paternal	Pathogenic	Panel sequencing	Incidental carrier finding
*RTTN*	c.6125C > A (*p*.Ser2042*)	Heterozygous	Maternal	Pathogenic	Panel sequencing	Incidental carrier finding

VUS, variant of uncertain significance; WGS, whole genome sequencing

**Fig. 1:**
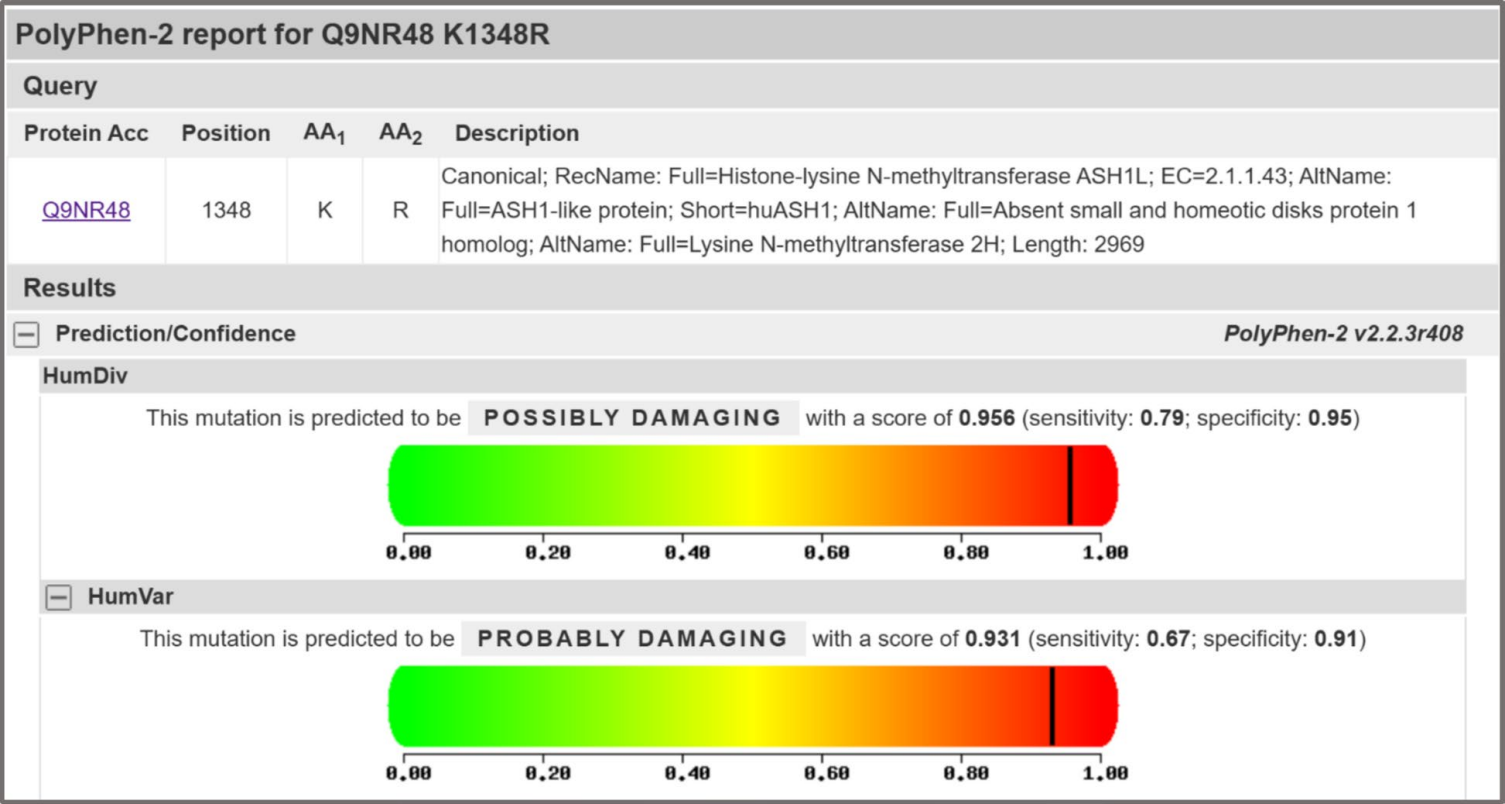
*In silico* prediction of the pathogenicity of the *ASH1L* *p*.Lys1348Arg variant. The output from the Polymorphism Phenotyping v2 (PolyPhen-2) analysis tool is shown. The tool predicts the functional impact of the *p*.Lys1348Arg amino acid substitution on the human ASH1L protein. The HumDiv model predicted the variant to be “possibly damaging” (score, 0.956), while the HumVar model, which is trained on a dataset of known disease-causing variants, predicted it to be “probably damaging” with a high confidence score of 0.931. Scores near 1.0 indicate a high likelihood that the variant is functionally deleterious

**Fig. 2:**
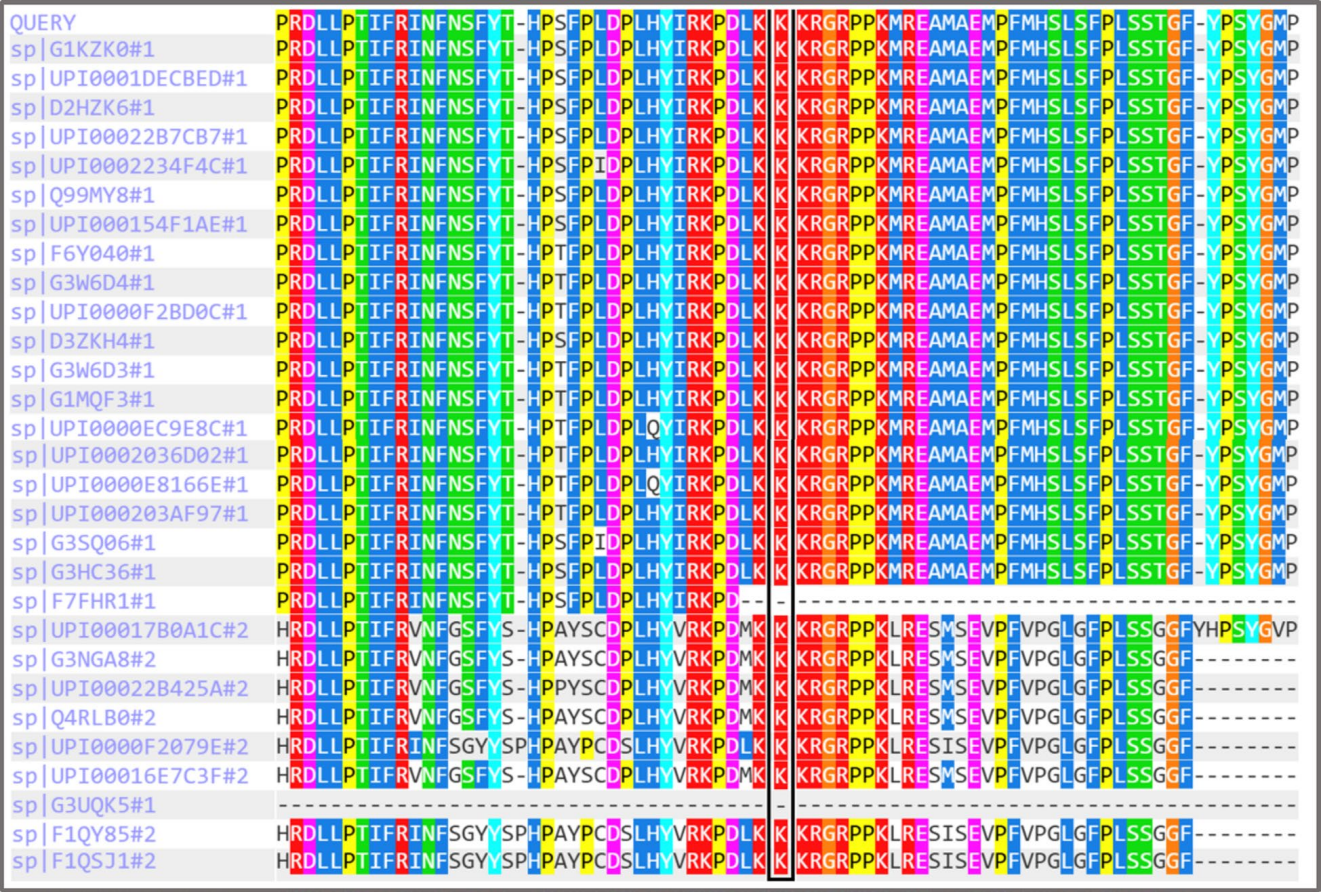
Evolutionary conservation of the *ASH1L* *p*.Lys1348 residue. This figure shows a multiple sequence alignment of the ASH1L protein sequence surrounding the variant of interest. The top row (“QUERY”) represents the human reference sequence, while the subsequent rows show the corresponding sequences from various other vertebrate species. The black box highlights position 1348. The lysine residue (“K”) at this position is shown to be highly conserved across all aligned species. This high degree of evolutionary conservation suggests that the *p*.Lys1348 residue is functionally critical and that substitutions at this site are likely to be deleterious

This variant was absent from population databases (ACMG criterion PM2) and its *de novo* status provided supporting evidence of pathogenicity. However, as it is located outside known functional domains and *in silico* predictors were inconclusive, it was classified as a VUS. To assess its potential functional impact, *in silico* predictive analysis was performed. The PolyPhen-2 tool [17] predicted the *p*.Lys1348Arg variant to be “probably damaging” with a score of 0.931 (HumVar model) (Fig. 1). Furthermore, analysis of multiple sequence alignments shows that the lysine residue at this position is highly conserved across species, suggesting it is functionally important (Fig. 2). Given the strong phenotypic overlap with IDD52, it was considered the most plausible explanation for his condition. WGS also identified other variants deemed unrelated to his primary presentation: a *de novo* VUS in *PDE4D*, a gene for acrodysostosis-2, a skeletal dysplasia the patient does not have; an incidental maternal intermediate *FMR1* allele; and carrier status for two autosomal recessive conditions.

## Discussion

### Molecular mechanism and functional implications

This report details a 6-year-old boy with a novel *de novo* missense *ASH1L* variant and a mild neurodevelopmental profile. His presentation overlaps with core IDD52 features, including global developmental delay, infantile hypotonia, ASD, and ADHD (Table 3). However, his borderline-to-average cognitive functioning is atypical, as most published cases report mild to severe ID. His specific cognitive split—average verbal versus impaired nonverbal skills—is a novel finding that expands the known phenotype. He also lacks seizures, a comorbidity seen in about 20–30% of individuals with IDD52 [4].

**Table 3:** Comparison of clinical features with the published ASH1L spectrum

Clinical domain	Feature	Proband (SP)	Published ASH1L cohorts (frequency/description)
Cognitive function	Intellectual disability	Borderline/average IQ	Present in ~100% of cases; typically, mild to severe ID [4].
	Specific profile	Significant verbal > nonverbal split	Not commonly described; expands known phenotype.
Behavior	Autism spectrum disorder	Yes, mild (level 1)	Common (~50–70%); severity varies [18].
	ADHD	Yes, combined type	Very frequent (~90%) [4].
	Obsessive traits	Yes (per TRF)	Common (~86%) [4].
Speech/language	Expressive/respective delay	Yes, primarily expressive delay	Speech delay is nearly universal; often severe [19].
Motor	Hypotonia	Yes, mild in infancy	Frequent early finding (~46%) [20].
	Coordination	Yes, mild motor incoordination	Common (~50–57% have motor delays/gait issues) [20].
Seizures	Epilepsy	No	Reported in a subset (~20–30%) [18].
Growth	Height/weight	Normal (40th–45th percentile)	Usually, normal [4].
	Head circumference (OFC)	Normal (25th–30th percentile)	Usually normal; microcephaly reported in some cases [4].
Dysmorphic features	Facial features	Mild (ear asymmetry, long face)	Mild, nonspecific features are common.
	Skin findings	2 café-au-lait macules	Not a known feature; likely incidental.
Congenital anomalies	Major malformations	No	Occasional (cardiac, GU, skeletal); not present in all [4].
Sleep	Sleep disturbances	Minor night awakenings only	Often reported as a significant issue [4].

Data for the proband are from comprehensive evaluation report. Literature data are synthesized from multiple sources. GU, genitourinary; IQ, intelligence quotient; OFC, occipital-frontal circumference

This case provides a critical counterpoint to the hypothesis that *ASH1L* missense variants uniformly cause more severe outcomes than truncating mutations [21]. The *p*.Lys1348Arg variant is located outside of well-characterized functional domains such as the catalytic SET domain [2]. A variant in a critical domain might exert a dominant-negative effect, whereas this variant’s location in a less constrained region, coupled with the conservative amino acid change (lysine to arginine), may result in a more subtle defect, such as partial loss of function. This could produce a milder clinical outcome more akin to haploinsufficiency. This case suggests that genotype–phenotype analyses must consider not just the variant type (missense versus LoF) but also its specific location and predicted functional impact.

### Diagnostic utility of whole genome sequencing

This case highlights the superior diagnostic yield of trio-WGS over targeted panels in complex neurodevelopmental disorders. Identifying the *ASH1L* variant, even as a VUS, provided the family a unifying diagnosis, ended their diagnostic odyssey, and allows for targeted anticipatory guidance (for example, seizure monitoring). Cost-effectiveness analyses support whole genome sequencing as first-tier testing in complex neurodevelopmental disorders, demonstrating reduced overall healthcare costs through decreased diagnostic odyssey duration and earlier intervention implementation [22].

### Study limitations and future directions

Publishing this detailed case contributes to the collective knowledge needed to reclassify VUSs, and the family was encouraged to join data-sharing platforms to help establish the variant’s pathogenicity. The primary limitation is the VUS classification of the *ASH1L* variant. While formally classified as a VUS due to its novelty, the case for its pathogenicity is substantially strengthened by several lines of evidence. In addition to its *de novo* occurrence and the strong phenotypic overlap, strong *in silico* predictions indicate a high likelihood of a damaging effect on protein function. The high evolutionary conservation of the lysine 1348 residue further suggests that its substitution would not be well tolerated. This collective evidence allows us to argue that the *p*.Lys1348Arg variant is likely pathogenic, even in the absence of functional studies.

As a single case report, generalizability is limited, and contributions from other genetic factors cannot be fully excluded. Additional *ASH1L* patients with similar variants would strengthen genotype–phenotype correlations and support clinical significance.

Long-term follow-up studies are needed to characterize developmental trajectories and intervention responses in *ASH1L*-related disorder. Establishing natural history data will inform prognostic discussions and optimize therapeutic approaches. Emerging therapeutic strategies targeting histone methyltransferase pathways may provide future treatment opportunities for patients with *ASH1L* variants [23].

## Conclusion

This report describes a 6-year-old boy with mild ASD and ADHD carrying a novel *de novo* missense *ASH1L* variant. His presentation, particularly his borderline-to-average intellectual ability, expands the known phenotypic spectrum of IDD52 and challenges oversimplified genotype–phenotype correlations. This case underscores the value of comprehensive genomic testing in resolving complex neurodevelopmental presentations that elude targeted panel testing. Continued reporting of well-phenotyped individuals is essential to reclassify VUSs and build a more sophisticated understanding of how variants in critical epigenetic regulators such as *ASH1L* shape human neurodevelopment.

## Data Availability

All data generated or analyzed during this study are included in this published article. Further details are available from the corresponding author on reasonable request.
